# Novel Genetic Analysis for Case-Control Genome-Wide Association Studies: Quantification of Power and Genomic Prediction Accuracy

**DOI:** 10.1371/journal.pone.0071494

**Published:** 2013-08-19

**Authors:** Sang Hong Lee, Naomi R. Wray

**Affiliations:** Queensland Brain Institute, The University of Queensland, Brisbane, Queensland, Australia; National Institute of Environmental Health Sciences, United States of America

## Abstract

Genome-wide association studies (GWAS) are routinely conducted for both quantitative and binary (disease) traits. We present two analytical tools for use in the experimental design of GWAS. Firstly, we present power calculations quantifying power in a unified framework for a range of scenarios. In this context we consider the utility of quantitative scores (e.g. endophenotypes) that may be available on cases only or both cases and controls. Secondly, we consider, the accuracy of prediction of genetic risk from genome-wide SNPs and derive an expression for genomic prediction accuracy using a liability threshold model for disease traits in a case-control design. The expected values based on our derived equations for both power and prediction accuracy agree well with observed estimates from simulations.

## Introduction

In the last five years, GWAS have been published for both quantitative traits (such as height [1], or blood markers [2]) and disease [3]. In order to assess the relative potential for success of these studies Yang et al. [4] provided an analytical method for comparison of power. For example, this method has been used to quantify that a sample of ∼50,000 schizophrenia cases and 50,000 controls is needed to afford the same power as the largest published GWAS of height (a total sample size of 180,000) [5].

Use of quantitative endophenotypes rather than binary traits has been proposed as a strategy to increase power in neuropsychiatric disorders [6]. Endophenotypes are measurable quantitative scores that are assumed to be associated with a continuous liability that underlies observed disease status, in which case the quantitative score may be more informative and powerful compared to binary responses. Of course, the true underlying liability would be the most informative although it is not observable. Recently, van der Sluis et al. [7] suggested a better use of phenotypic information in GWAS of psychiatric disorders measured in population cohorts. Rather than using binary responses of affected/non-affected they considered the use of continuous scores from diagnostic instruments. They showed that binary responses based on clinical cut-off criteria decreased power dramatically compared to the use of sum scores of item responses from the diagnostic instrument. The authors recommended that continuous quantitative responses such as sum scores of item responses should be used in psychiatry disorder GWAS, where possible. The study by van der Sluis et al. [7] compared scenarios by simulation and was based on population samples. Here, we provide an analytical method to calculate power in different scenarios with both population and case control samples.

Another potential use of data collected in GWAS is the prediction of genetic risk. Genomic-enabled prediction is a potentially powerful tool to identify individuals at higher risk of disease [3], [8]. Undoubtedly, prediction accuracy plays a crucial role in a successful clinical application for genetic risk prediction of disease, and several studies have evaluated the predictive ability [9], [10], [11]. Daetwyler et al. [12] derived a theoretical accuracy for predicting genetic risk from genome-wide SNPs, based on least squares methodology. Many studies have used their formula, which works well for quantitative traits. However, in simulation studies their formula for case-control traits underpredicted the true accuracy (Table 4 of Daetwyler et al. [12]).

In this study, we address two issues relevant for the design case-control GWAS, power and genomic prediction accuracy. First, we derive analytically, in a unified framework, the power of GWAS when using population or ascertained case-control samples with binary as well as quantitative responses. Secondly, we derive genomic prediction accuracy based on the 0,1 observed scale, and transform it to the liability scale using a liability threshold model for disease traits in population [13] and in case-control samples [14]. The expected values based on our derived equations and the average of observed estimates from simulation agree well.

## Materials and Methods

### Power

Given a specified critical value for significance, power of a given association study design can be derived from the non-centrality parameter (NCP, λ) of a **χ** ^2^ test of association. Following methods of Yang et al. [4] we derive the NCP for five different experimental designs:, i.e. quantitative responses in population (QT_POP) (1), binary responses in population (BT_POP) (2), binary responses in ascertained case-control samples (BT_CC) (3), quantitative responses in ascertained case-control samples (QT_CC) (4) and samples of both ascertained cases and controls in which quantitative responses are available in the cases only (QB_CC) (5). The derived NCP for BT_CC, QT_CC and QB_CC are novel. Following Yang et al. [4],

*1) NCP for quantitative responses in population samples, 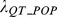
*



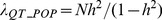
(1)where N is the total number of sample, 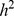 is the proportion of variance explained by a single genetic marker or set of markers, i.e. multi locus association tests [15], [16].

*2) NCP for binary traits in population samples*



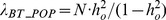
(2)where 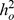 is the proportion of variance explained by a genetic marker or set of markers on the observed scale, and 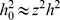 [13], where *z* is the height of the normal curve truncating the proportion *K*, where *K* is the proportion of the population that are cases.

*3) NCP for binary responses in ascertained case-control samples, 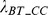
*

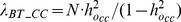
(3)where 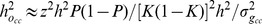, [14], [17], with *P* the proportion of cases in the case control sample and 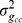 the genetic variance in the case-control sample inflated relative to the population sample as a result of the ascertainment process [12], such cases are over-represented compared to the population sample. When 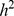 and 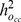 is small, (3) can be approximated and simplified as 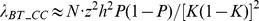, which agrees with the derivation based on the relative risk and multiplicative model by Yang et al. [4].

*4) NCP for quantitative responses in ascertained case-control samples, 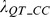
*

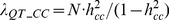
(4)where 
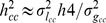
, where 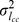 is the variance of disease liability [18]. This equality is derived from quantitative genetic theory [18] in the following way. Firstly,
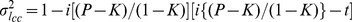
(5)where *i* is the mean liability in cases and t is the threshold on the normal distribution which truncates the proportion of disease prevalence K, and from Daetwyler et al. [12]



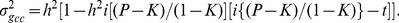
(6)In a similar manner, the inflated variance due to non-genetic effects is,
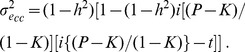
(7)


The covariance between disease liability and genetic values in an ascertained case-control sample is
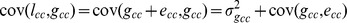
where 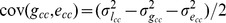.

Therefore, from (5), (6) and (7),
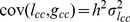



The regression coefficient of *l_cc_* on *g_cc_* is
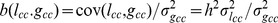



Finally, the proportion of variance attributable to the SNPs or set of SNPs of a quantitative response in an ascertained case-control sample can be obtained as the squared regression coefficient multiplied by the genetic variance in the case-control sample and scaled by the variance of disease liability in the case control sample, i.e.
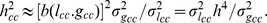



*5) NCP for quantitative responses for cases only in ascertained case-control samples,* 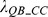


When underlying continuous quantitative responses are available only for cases in the ascertained case-control sample, i.e. the recorded values follow a mixture distribution of zero for controls and truncated normal distribution for cases. An example may be a GWAS of major depressive disorder in which cases are recorded for a quantitative severity score, whereas controls have not been scored. In this situations,
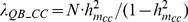
(8)where 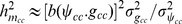, which is explained as follows.

The variance of the mixed zero and truncated normal values in an ascertained case-control sample is,
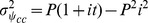
where *i* and *t* are the same as defined above. There is an assumption here is that the quantitative trait is the phenotypic liability.

The covariance between 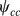 and *g_cc_* in an ascertained case-control sample is,
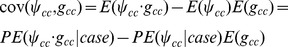
where 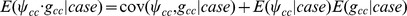,



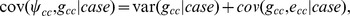
and,



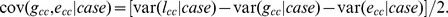



From the equations above, regression coefficient of 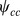 on g_cc_ can be derived analytically as,
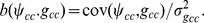



Therefore, the proportion variance attributable to the variance in the SNPs from mixed zero and quantitative response in an ascertained case-control sample (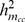) can be expressed as above under (8). The power for this mixed 0 and truncated normal responses is very similar to that for BT_CC (results not shown).

### Genomic prediction accuracy

#### Normal quantitative traits

For a quantitative trait, *β_j_* is the random allelic substitution effect of the *j*th single nucleotide polymorphism (SNP). Following Daetwyler et al. [12], prediction error variance for the *j*th SNP effect is
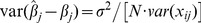
(9)where 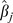 is the estimate of the true regression *β_j_* of the phenotype on the *j*th SNP genotype, *x_ij_*  = 0, 1 or 2 for the *i*th individual, *N* is the number of individual records and *σ^2^* is the residual variance.

Assuming a phenotypic variance of one, the genetic variance (var(*g*)) explained by the set of *M* SNPs is *h^2^*. Following Daetwyler et al. [12], the estimated genetic variance explained by the *M* SNPs in the predictor (var(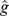)) is a function of the *h^2^*, *M*, the number of records (*N*) and the residual variance (*σ^2^*) as
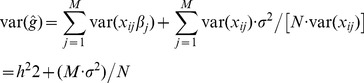



The squared correlation coefficient between the true and estimated genetic value is the ratio of the true genetic variance over the estimated genetic variance [12] as
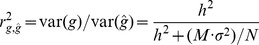
where the residual variance would be approximated as *σ^2^* = 1 (phenotypic variance) as in Daetwyler et al. [12]. With *τ* defined as the ratio of the number of samples (*N*) over the number of SNPs (*M*), the accuracy can also be written as [12]



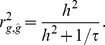



#### Disease traits in population sample

In binary disease traits, with *σ^2^* approximated as *σ^2^* = *K(1–K)* (i.e. binomial phenotypic variance for a disease with population prevalence of *K*), the prediction error variance for the *j*th SNP effect can be written as
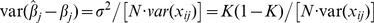
where *β* is allele substitution effect on the 0, 1 observed scale and 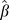 is the estimated *β* from regression of the 0,1 discrete phenotypes on SNP coefficients. The estimated genetic variance on the observed scale of the SNP predictor (var(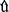)) is a function of the genetic variance on the observed scale (var(*u*) or 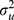), the number of SNPs, the number of records and the residual variance as



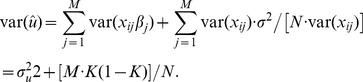



The squared correlation coefficient between the true and estimated genetic values is
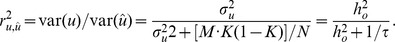



Because genetic variance as a proportion of phenotypic variance on the observed scale can be transformed from that on the liability scale as 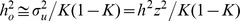 [13], prediction accuracy can be re-expressed as
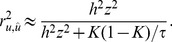
(10)


Equation (10) here is the same as equation (6) in Daetwyler et al. [12].

#### Disease traits in ascertained case-control study

Ascertainment in case-control samples often results in over-representation of cases compared to the case prevalence in the population. The variance of the explanatory variable is inflated by a factor of 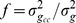 [12], [17]. The term, 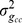, is the inflated genetic variance due to ascertainment in case-control sample [12]. Therefore, the inflated explanatory variable for the *j*th SNP can be written as 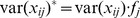. Then, the prediction error variance for the *j*th SNP effect can be expressed as
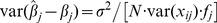
where *β* is allele substitution effect on the 0, 1 observed scale and 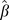 is estimated *β* from regression of the 0,1 discrete phenotypes on SNP coefficients in the case-control sample. The estimated genetic variance on the observed scale in a case-control design can be derived as



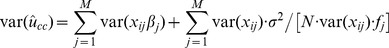
where var(*x_ij_β_j_*) is the genetic variance on the observed scale due to the *j*th SNP effect transformed to the liability scale [14], [17] 
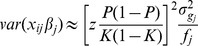
. With a sufficient number of causal SNPs (>∼20), the residual variance is approximated as *σ^2^* = *P*(1–*P*) (i.e. the binomial phenotypic variance in a case-control sample where the proportion of cases is *P*), and the value for *f* is close to 1 (i.e. a small fraction of genetic variance has a negligible inflation). Therefore, the genetic variance in a case-control sample is



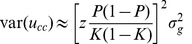
and, the estimated genetic variance in a case-control sample is approximately



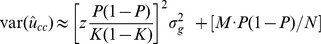



The squared correlation coefficient between the true and estimated genetic values is
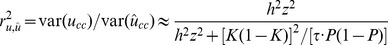
(11)


Equation (11) differs from equation (9) of Daetwyler et al. [12], i.e.
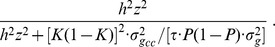



For binary traits, area under the receiver-operator characteristic curve (AUC) is a useful statistic for the genomic prediction accuracy [19], [20]. A relationship between the correlation coefficient and AUC has been shown in previous studies [11], [20].

## Simulation Study

### Power

In order to check the analytically derived equations of NCP for BT_POP, BT_CC, QT_POP and QT_CC, we carried out a simulation study. Individual genetic values (*g*) were simulated from an additive multilocus model of *M* = 100 independent SNPs with equal allele effects and allele frequency of 0.5. Residual values (*e*) were independently generated from a random normal distribution with a mean of zero and variance of 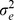. The value of 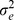 was set relative to 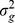 so that the desired proportion of variance explained by the markers, *h^2^* was obtained. We simulated *h^2^* of 0.01, 0.05 and 0.1 so that each SNP explained 0.0001, 0.0005 and 0.001 of the phenotypic variance (Table 1). Liability phenotypes for each individual were simulated as *y*  =  *g* + *e*. Affected individuals were those with liability phenotype that exceeded a threshold determined by population prevalence. The numbers of cases and controls in the sample was 2000. The values for population prevalence were varied as *K* = 0.1, 0.01 or 0.001. The proportion of cases was *P* = *K* in simulations of population sample and *P* = 0.5 in simulations of case-control sample where cases were over-sampled by a factor (1–*K*)/*K*. In population or case-control sample, we used both binary (BT_POP or BT_CC) and quantitative responses (QT_POP or QT_CC). We conducted 100 replicates for each simulation scenario, therefore 10000 association tests were carried out. Power was calculated as the proportion of the 10000 association tests in which the association p-value less than 0.05 and was compared to power calculated from the NCP using a function in R package, i.e. power  = 1– pchisq (T, 1, ncp  =  NCP) where T is the normal distribution threshold corresponding to the significance level 0.05.

**Table 1:** Expected power for an association study from the derived equations and observed averaged power from simulation.

	BT_POP		BT_CC^a^		QT_POP		QT_CC^a^	
*h^2^*	Exp	Obs (SE)	Exp	Obs (SE)	Exp	Obs (SE)	Exp	Obs (SE)
N = 2000, *K* = 0.1								
0.0001	0.058	0.053 (0.002)	0.072	0.072 (0.003)	0.073	0.075 (0.003)	0.082	0.083 (0.003)
0.0005	0.090	0.086 (0.003)	0.164	0.163 (0.004)	0.170	0.172 (0.004)	0.218	0.221 (0.004)
0.001	0.131	0.130 (0.003)	0.281	0.286(0.005)	0.293	0.294 (0.005)	0.386	0.386 (0.005)
N = 2000, *K* = 0.01								
0.0001	0.052	0.057 (0.002)	0.092	0.092 (0.003)	0.073	0.075 (0.003)	0.105	0.102 (0.003)
0.0005	0.058	0.057 (0.002)	0.270	0.267(0.004)	0.170	0.169 (0.004)	0.333	0.329 (0.005)
0.001	0.067	0.066 (0.002)	0.478	0.474 (0.005)	0.293	0.295 (0.005)	0.579	0.574 (0.005)
N = 2000, *K* = 0.001								
0.0001	0.050	0.042 (0.002)	0.117	0.117(0.003)	0.073	0.075 (0.003)	0.130	0.132 (0.003)
0.0005	0.051	0.052 (0.002)	0.392	0.387 (0.005)	0.170	0.176 (0.004)	0.451	0.451 (0.005)
0.001	0.053	0.052 (0.002)	0.664	0.657 (0.005)	0.293	0.296 (0.005)	0.738	0.733 (0.004)

*h* ^2^: variance explained by the locus.

a: in case-control samples, 50% of the sample are cases, P = 0.5.

Exp: Expected power based on NCP derived from equation (1)∼(4).

Obs: Averaged power over 10000 replicates of simulation.

SE: Empirical standard error over 10000 replicates.

### Genomic prediction accuracy

Simulations were carried out to verify the validity of equations (10) and (11). In a simulation study, individual genetic values (*g*) were simulated from an additive multilocus model with equal allele effects (allele frequency of ∼0.5) and residual values (*e*) independently generated from a random normal distribution with a mean of zero and variance of 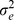. The value of 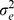 was set relative to 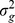 so that the desired proportion of variance explained by the markers, *h^2^* was obtained. Liability phenotypes for each individual were simulated as *y*  =  *g* + *e*. Affected individuals were those with liability phenotype that exceeded a threshold determined by population prevalence. Population prevalences of *K* = 0.001, 0.01, 0.1, 0.2 and 0.5 were used with *N* = 2000 and *M* = 2000. To vary *τ* =  *N*/*M*, *N* = 2000 and *M* = 400 were used for *τ* = 5, and *N* = 100 and *M* = 5000 were used for *τ* = 0.02. Following Daetwyler et al. [12], allele substitution effects (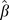) were estimated using a regression analysis for each simulated SNP. As a validation set, a second sample of individuals was generated based on the same genetic parameters as in the original population. Empirical prediction accuracy can be obtained by correlating the true genetic values (*g*) and estimated genetic values 
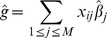
 in the validation set.

## Results

### Power

The power of association tests observed in simulation and expected from theory agreed well under a range of scenarios (Table 1). Whether using lower or higher values of disease prevalence *K*, there was an excellent agreement between the observed and expected power with a small empirical standard error. When using a higher variance explained by each locus (*h^2^*), although the empirical standard error increased slightly, the observed value also agreed well with the expected value (Exp and Obs in Table 1).

**Figure 1:**
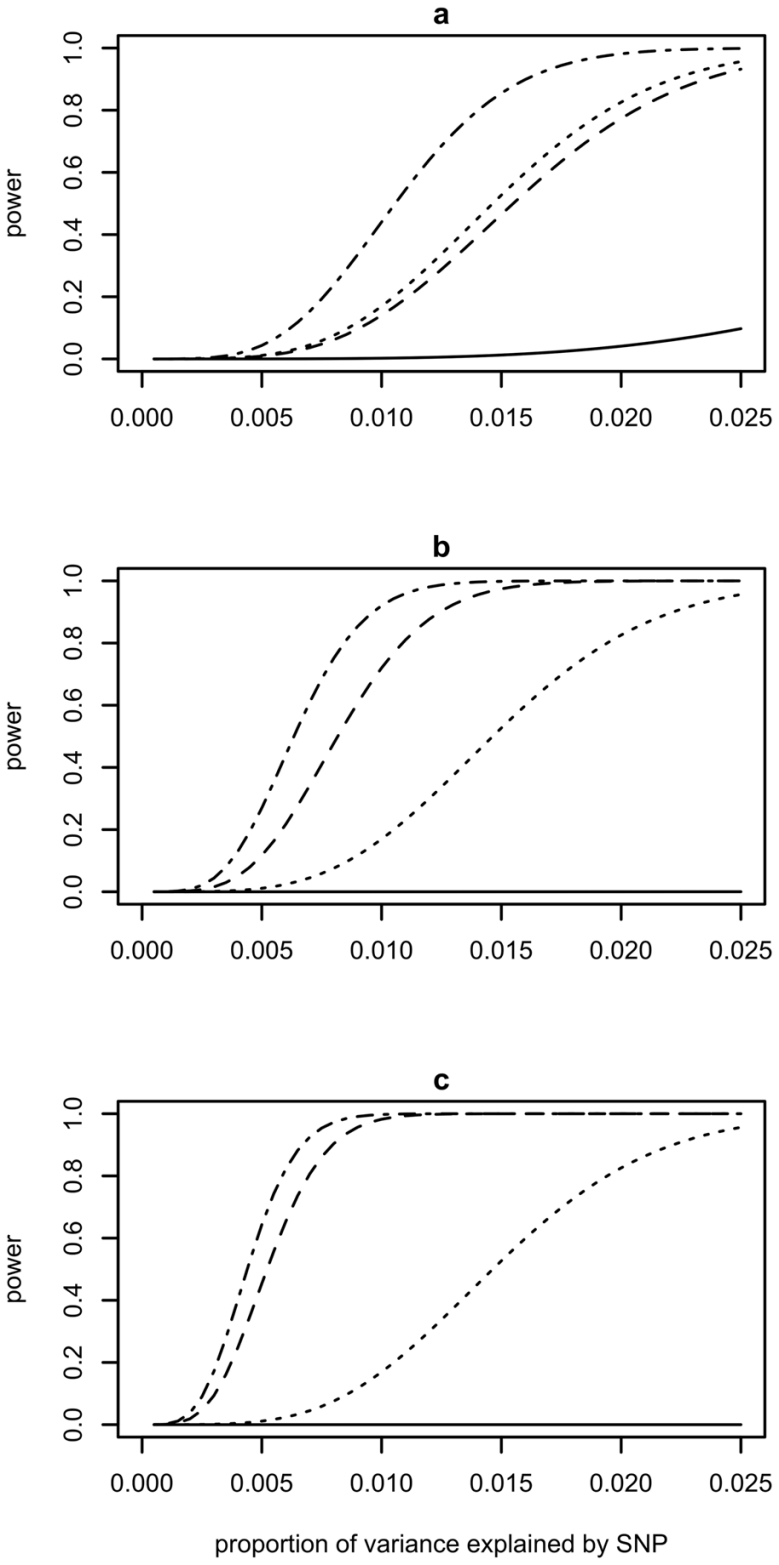
Power derived for QT_POP (dotted line), BT_POP (solid line), BT_CC (dashed line) and QT_CC (dot-dashed line) when using population prevalence *K* = 0.1 (a), *K* = 0.01 (b) or *K* = 0.001 (c) assuming the same total sample size N = 2000 and a critical significance threshold of 5×10^−8^.

In Figure 1, values for the power based on NCP derived from equations (1)∼(4) were plotted against variance explained by SNPs (i.e., *h^2^*). Generally, the power increases when the variance explained by SNPs increases, and when the ascertained case-control design is used. For BT_POP, the power decreases as *K* decreases, reflecting the smaller number of cases in a given population sample. For QT_POP, the power is, of course, constant across a–c in Figure 1. When using an ascertained sample (BT_CC or QT_CC), the power increases as the value for *K* decreases, which reflects the greater over-sampling of cases with lower *K* for the same sample size and hence the difference in mean liability between cases and controls increases. There is a moderate difference between BT_CC and QT_CC when using population prevalence *K* = 0.1 (a in Figure 1). The difference between BT_CC and QT_CC becomes smaller with lower values for *K* (b and c in Figure 1).

### Genomic prediction accuracy

The expected accuracies predicted from equation (11) agreed well with the observed average of estimates from simulation for all simulation scenarios for both population and ascertained case-control samples. In Table 2, disease prevalence *K* varies, in Table 3 proportion of variance explained by SNPs *h^2^* varies and in Table 4, values for *τ* = *N*/*M* vary. For comparison, we list also the predicted accuracies for case-control samples provided in Daetwyler et al. [12]. As shown in their Table 4, their formula underestimates prediction accuracy particularly when disease prevalences are low (Table 2) and *h^2^* are high (Table 3). We also tested the prediction accuracy with allele effects sampled from a normal or an exponential distribution. The results from these alternative distributions of allele effects were not much different from the main results (results not shown). This agrees with Daetwyler et al. [12] in that the derived prediction accuracy is robust to distributional assumption for allele effects.

**Table 2:** Prediction accuracy for a disease with population or case-control samples when true proportion of variance explained by the set of SNPs on the liability scale is 0.5, *τ* =  N/M is 1 for different disease prevalences.

Prevalence	Population		Case-Control		
	Exp1	Est (se)	Exp2	Exp3	Est (se)
0.001	0.075	0.063 (0.004)	0.628	0.766	0.767 (0.002)
0.01	0.186	0.183 (0.003)	0.594	0.689	0.690 (0.002)
0.1	0.382	0.377 (0.003)	0.533	0.568	0.570 (0.002)
0.2	0.444	0.438 (0.003)	0.511	0.526	0.529 (0.003)
0.5	0.491	0.487 (0.003)	0.491	0.491	0.487 (0.003)

Exp1: Expected value from equation (2) or equation (6) of Daetwyler et al. (2008).

Exp2: Expected value from equation (9) of Daetwyler et al (2008).

Exp3: Expected value from equation (3).

Est: Average of estimates from 100 replicates.

se: Empirical standard error over 100 replicates.

Proportion of cases in case-control study is P = 0.5.

**Table 3:** Prediction accuracy for a disease with population or case-control samples when prevalence is 0.01, *τ* =  N/M is 1 for diseases with different *h^2^*.

*h^2^*	Population		Case-Control		
	Exp1	Est (se)	Exp2	Exp3	Est (se)
0.1	0.084	0.087 (0.004)	0.371	0.392	0.395 (0.003)
0.5	0.186	0.183 (0.003)	0.594	0.689	0.690 (0.002)
0.9	0.246	0.243 (0.003)	0.653	0.787	0.787 (0.001)

Exp1: Expected value from equation (2) or equation (6) of Daetwyler et al. (2008).

Exp2: Expected value from equation (9) of Daetwyler et al (2008).

Exp3: Expected value from equation (3).

Est: Average of estimates from 100 replicates.

se: Empirical standard error over 100 replicates.

Proportion of cases in case-control study is P = 0.5.

**Table 4:** Prediction accuracy for a disease with population or case-control samples when true proportion of variance explained by the set of SNPs on the liability scale is 0.5, prevalence is 0.01 and *τ* =  N/M varies.

*τ* = N/M	Population		Case-Control		
	Exp1	Est (se)	Exp2	Exp3	Est (se)
0.02	0.027	0.028 (0.003)	0.104	0.133	0.124 (0.004)
1	0.186	0.183 (0.003)	0.594	0.689	0.690 (0.002)
5	0.390	0.389 (0.004)	0.731	0.905	0.905 (0.001)

Exp1: Expected value from equation (2) or equation (6) of Daetwyler et al. (2008).

Exp2: Expected value from equation (9) of Daetwyler et al (2008).

Exp3: Expected value from equation (3).

Est: Average of estimates from 100 replicates.

se: Empirical standard error over 100 replicates.

Proportion of cases in case-control study is P = 0.5.

## Discussion

Firstly, we provide analytical derivations in a unified framework to quantify the power of GWAS when using population or ascertained case-control samples with binary responses or quantitative responses. The derived equations were validated in a simulation study, showing that expected values from the equations and observed values from simulations agreed well. Secondly, following Daetwyler et al. [12], we derive an expression genomic prediction accuracy based on the 0,1 observed scale, and transformed it to that on the liability scale using a liability threshold model for disease traits in population [13] and in case-control samples [14]. Compared with Daetwyler et al. [12], our derivation agrees for population samples, but is more accurate for case-control samples.

The Genetic Power Calculator [21] is commonly used for calculation of power is genetic association studies. The calculator is based on theoretical derivation [22], [23] of a single locus model with required parameters of allele frequency and its effect size (e.g. relative risk or odds ratio in binary responses). However, our derivations and application did not require those parameters (see equation (3), (4) and (8) and Appendix S1 and S2 for application) because our derivations are based on variance explained by a locus, and many combinations of allele frequency and effect size can generate the same variance explained. Our framework easily accommodates power of association of multiple loci because we use a single parameter for the total variance that is generated by any number of loci. Applications of multiple loci association GWAS have been published recently [24], [25]. In practice, the power to detect causal variants may not exactly agree with our analytical derivations because of unknown parameters such as linkage disequilibrium among variants and distribution of effect size that alter the effective number of tests. We recommend that such unknown parameters should be carefully considered in applying power calculation.

Recently, Dudbridge [11] proposed a comprehensive study about power and predictive accuracy of polygenic scores. Our equation (11) and Dudbridge's equation (13) [11] are analogous to each other. However, Dudbridge used his equation (13) with a heuristic justification from simulations. We analytically derived equation (11) based on a liability threshold model and gave a reasonable explanation why *f_j_* is approximated as 1.

Lastly, van der Sluis et al. [7] quantified by simulation the power lost in genetic association analyses of population samples measured for quantitative endophenotypes but analysed with a dichotomous case-control score. Our analytical derivations for such scenarios allow easy generalization of their results to the design of new studies.

## Supporting Information

Appendix S1 R code for the power derivations described in the paper.
(DOC)

Appendix S2 R code for the prediction accuracy derivations described in the paper.
(DOC)
