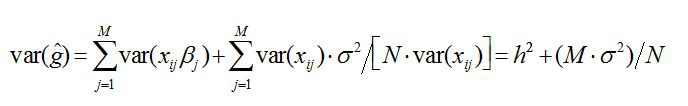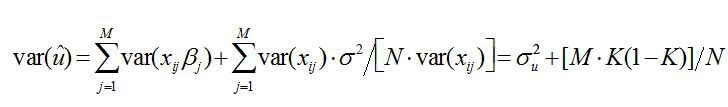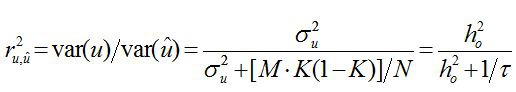# Correction: Novel Genetic Analysis for Case-Control Genome-Wide Association Studies: Quantification of Power and Genomic Prediction Accuracy

**DOI:** 10.1371/annotation/0b29c9c7-a86d-4e0f-bbb8-5c29b16e2884

**Published:** 2013-08-28

**Authors:** Sang Hong Lee, Naomi R. Wray

There were errors in the equation directly following Equation 9, and the two equations directly preceding Equation 10. Correct versions of all three equations are available in that order below.