# Supplementary material for: Novel Genetic Analysis for Case-Control Genome-Wide Association Studies: Quantification of Power and Genomic Prediction Accuracy
Source: PLoS One. 2013 Aug 19;8(8):e71494. doi: 10.1371/journal.pone.0071494 (PMC3747270; doi:10.1371/journal.pone.0071494)
Supplement: Appendix S1 — R code for the power derivations described in the paper. (DOC) [file pone.0071494.s001.doc]

**Appendix S1: R code for the power derivations described in the paper**

ccpower = function (h2,N,k,p,spv) {

cat("\n")

cat("*************************************************************","\n")

cat("usage : ccpower(h2,N,k,p,spv)","\n")

cat("h2 : proportion of variance explained by SNPs on the liability scale","\n")

cat("N : sample size","\n")

cat("k : population prevalence","\n")

cat("p : proportion of cases in case-control sample","\n")

cat("spv : p-value (significance level) to get power given NCP","\n")

cat("*************************************************************","\n")

cat("\n")

thd=-qnorm(k) #threshold

zv=dnorm(thd) #height at the normal curve

iv=zv/k #mean liability for cases

iv2=-iv*k/(1-k) #mean liability for controls

cv=(k*(1-k))^2/(zv^2*p*(1-p)) #the spread sheet

theta=iv*((p-k)/(1-k))*(iv*((p-k)/(1-k))-thd)

vgcc=h2*(1-h2*theta) #g variance on the liability in CC

h2o=h2/(cv-h2*theta*cv)

h2c=(1-theta)*(h2^2/(h2*(1-h2*theta))) #from power_note.doc

#for QB_CC (control 0 and cases have continous value)

vl1=1-iv*(iv-thd) #var(y*|y=1)

vg1=h2-h2*iv*(h2*iv-h2*thd) #var(g|y=1)

ve1=(1-h2)-(1-h2)*iv*((1-h2)*iv-(1-h2)*thd) #var(g|y=1)

cov_ge1=(vl1-vg1-ve1)/2 #cov(g,e|y=1)

cov_yg=p*(vg1+cov_ge1+iv*h2*iv) - p*iv* (p*iv*h2 + (1-p)*iv2*h2) #cov(y,g)

vl= p*(1+iv*thd) - p^2*iv^2 #var(y*)=p(1=it) - p^2i^2

h2c2=(cov_yg/vgcc)^2*vgcc / vl

vu=h2o*p*(1-p) #g varaince on the observed scale in BT_CC

#NCP

ncp1=N*h2/(1-h2)

ncp2=N*h2o/(1-h2o)

ncp3=N*h2c/(1-h2c)

ncp5=N*h2c2/(1-h2c2)

alpha=qchisq(1-spv,1)

pow1=1-pchisq(alpha,1,ncp=ncp1)

pow2=1-pchisq(alpha,1,ncp=ncp2)

pow3=1-pchisq(alpha,1,ncp=ncp3)

pow5=1-pchisq(alpha,1,ncp=ncp5)

mat=matrix(0,4,3)

mat[1,1]=h2

mat[2,1]=h2o

mat[3,1]=h2c

mat[4,1]=h2c2

mat[1,2]=ncp1

mat[2,2]=ncp2

mat[3,2]=ncp3

mat[4,2]=ncp5

mat[1,3]=pow1

mat[2,3]=pow2

mat[3,3]=pow3

mat[4,3]=pow5

colnames(mat)=c("h2","NCP","power")

rownames(mat)=c("QT_pop:","BT_CC:","QT_CC:","QB_CC:")

out <- signif(mat,digits=3)

print(out,quote=F,col.name=T,row.name=T)

#example

#ccpower(h2=0.005,N=4000,k=0.01,p=0.5,spv=0.00000005)

}
