# Supplementary material for: Novel Genetic Analysis for Case-Control Genome-Wide Association Studies: Quantification of Power and Genomic Prediction Accuracy
Source: PLoS One. 2013 Aug 19;8(8):e71494. doi: 10.1371/journal.pone.0071494 (PMC3747270; doi:10.1371/journal.pone.0071494)
Supplement: Appendix S2 — R code for the prediction accuracy derivations described in the paper. (DOC) [file pone.0071494.s002.doc]

**Appendix S2: R code for the prediction accuracy derivations described in the paper**

ccgpa = function (h2,N,M,k,p) {

cat("\n")

cat("*************************************************************","\n")

cat("usage : ccgpa(h2,N,M,k,p)","\n")

cat("h2 : proportion of variance explained by predictor on the liability scale","\n")

cat("N : sample size","\n")

cat("M : number of SNPs","\n")

cat("k : population prevalence","\n")

cat("p : proportion of cases in case-control sample","\n")

cat("*************************************************************","\n")

cat("\n")

thd=-qnorm(k) #threshold

zv=dnorm(thd) #height at the normal curve

iv=zv/k #mean liability for cases

iv2=-iv*k/(1-k) #mean liability for controls

cv=(k*(1-k))^2/(zv^2*p*(1-p)) #the spread sheet

theta=iv*((p-k)/(1-k))*(iv*((p-k)/(1-k))-thd)

lamda=N/M

vgcc=h2*(1-h2*theta) #g variance on the liability in CC

h2o=h2/(cv-h2*theta*cv)

r2=h2*zv^2/(h2*zv^2+(k*(1-k))^2/(lamda*p*(1-p))) #derivation in this study

D_r2=h2*zv^2/(h2*zv^2+(k*(1-k))^2*vgcc/(lamda*p*(1-p)*h2)) #derivation in Datewyler et al. 2008

mat=matrix(0,2,2)

mat[1,1]=r2

mat[1,2]=r2^.5

mat[2,1]=D_r2

mat[2,2]=D_r2^.5

colnames(mat)= c("variance (r^2)","accuracy (r)")

rownames(mat)= c("Eq (11) in this study:","Daetwyler derivation:")

out <- signif(mat,digits=4)

print(out,quote=F,col.name=F,row.name=F)

}
